# The garden as a laboratory: the role of domestic gardens as places of scientific exploration in the long 18th century

**DOI:** 10.1179/0079423614Z.00000000054

**Published:** 2014-06

**Authors:** CLARE HICKMAN

## Abstract

Eighteenth-century gardens have traditionally been viewed as spaces designed for leisure, and as representations of political status, power and taste. In contrast, this paper will explore the concept that gardens in this period could be seen as dynamic spaces where scientific experiment and medical practice could occur. Two examples have been explored in the pilot study which has led to this paper — the designed landscapes associated with John Hunter’s Earl’s Court residence, in London, and the garden at Edward Jenner’s house in Berkeley, Gloucestershire. Garden history methodologies have been implemented in order to consider the extent to which these domestic gardens can be viewed as experimental spaces.

## Abstract (fr)

**Le jardin comme laboratoire: le rôle des jardins domestiques comme place d’exploration scientifique au cours du XVIII^e^ siècle**

Les jardins du XVIII^e^ siècle ont traditionnellement été considérés comme des espaces conçus pour le loisir, et comme représentations du goût, du pouvoir et du statut politiques. Cet article explore au contraire le concept de jardin en tant qu’espace dynamique où peuvent s’exercer expérimentation scientifique et pratique médicale. Cet article rapporte l’étude pilote de deux exemples: les paysages conçus pour la résidence du John Hunter à Earls Court, à Londres, et le jardin de la maison de Edward Jenner à Berkeley, Gloucestershire. Les méthodologies sur l’histoire du jardin ont été mises en place afin d’évaluer à quel point ces jardins domestiques peuvent être considérés comme espaces d’expérimentation.

## Abstract (de)

**Der Garten als Laboratorium: die Rolle häuslicher Gärten durch wissenschaftliche Nutzung während des langen 18. Jahrhunderts**

Gärten des 18. Jahrhunderts wurden traditionellerweise als Gebiete zur Erholung betrachtet, außerdem repräsentierten sie politischen Status, Macht und Geschmack. Im Gegensatz dazu verfolgt diese Studie die Ansicht, dass Gärten dieser Periode auch als dynamische Flächen angesehen werden könnten, in denen wissenschaftliche Experimente und medizinische Versuche praktiziert wurden. Zwei Beispiele sind in einer Pilotenstudie untersucht worden und führten zu diesem Artikel: das Landschaftsdesign der Earls Court Residenz des John Hunter in London, und der Garten des Edward Jenner in Berkeley, Gloucestershire. Methodologische Gartengeschichte wurde angewendet, um festzustellen, in welchem Grade diese Gärten hinsichtlich experimenteller Raumgestaltung angesehen werden können.

## Abstract (it)

**Il giardino come laboratorio: il ruolo dei giardini domestici come luoghi di ricerca scientifica durante il lungo XVIII secolo**

Tradizionalmente, i giardini del XVIII secolo sono visti come spazi disegnati per il tempo libero, e come proiezione dello status politico, del potere, e del gusto. Questo contributo vuole invece esplorare l’idea secondo la quale, in questo periodo, i giardini potrebbero essere visti come spazi dinamici, luoghi dove gli esperimenti scientifici e la pratica medica possono prendere forma. Lo studio-guida che ha portato a questo lavoro analizza due esempi: l’architettura del paesaggio associata alla residenza di John Hunter, Earl’s Court a Londra, e i giardini presso la casa di Edward Jenner a Berkeley, nel Gloucestershire. Le metodologie proprie della storia dei giardini sono state implementate in modo da prendere in considerazione fino a che punto questi giardini domestici possano essere considerati come spazi sperimentali.

## Abstract (es)

**El jardín como un laboratorio: el papel de los jardines domésticos como lugares de la exploración científica en el siglo XVIII**

Los jardines del siglo XVIII han sido vistos tradicionalmente como espacios diseñados para el ocio además de reflejar el estatus político, poder y buen gusto. En este artículo, por otro lado, se explora la noción de que los jardines también podrían haber sido durante este período espacios dinámicos donde se podrían desarrollar experimentos científicos y prácticas médicas. A modo de un estudio preliminar que ha impulsado este artículo se estudiaron dos ejemplos: los paisajes diseñados asociados con la residencia Earl’s Court de John Hunter, Londres, y el jardín de la casa de Edward Jenner en Berkeley, Gloucestershire. Se han utilizado metodologías que estudian la historia del jardín con el fin de considerar hasta qué punto estos jardines domésticos pueden considerarse como espacios experimentales.

## INTRODUCTION

In garden history terms, interactions with gardens, particularly those of the 18th century, have traditionally been described in relation to aesthetics, pleasure-seeking and displays of power,^1^ rather than as active sites for the production of knowledge, prefiguring the modern scientific laboratory. The intention of this article is to add to the growing literature which aims to broaden our understanding of gardens beyond the traditional focus of elite landscapes. The use of garden history methodology, with its focus on the close reading of individual sites through site walking (where possible), maps and primary archival research, can complement and add new dimensions to our understanding of the role and use of domestic gardens. Garden history, with its interest in space, place and material cultures, speaks to recent trends in other historical fields, and of course geography and archaeology. It also forms part of a wider movement which seeks to further our historical understanding through an analysis centred on material culture — in this case, physical landscape features.

This approach corresponds to the growing interest in the development and practice of science beyond that of the 19th-century construct of the laboratory.^2^ However, unlike recent work in this area by Robert Kohler, Simon Naylor and others, this article will focus on designed spaces rather than the field and, therefore, also seek to redefine the garden as a ‘liminal’ space which exists between the wilder ‘field’ and the more managed and ideally placeless ‘laboratory’. This approach will build on the research being conducted, predominantly by historical geographers, on the relationship between space, place and science. David Livingstone’s *Putting Science in its Place* included botanic gardens as part of his investigation into the relationship between scientific practice and place,^3^ and Paul Elliott has described the garden of Erasmus Darwin in relation to the man’s scientific and medical interests.^4^ Research has also been conducted on the scientific use of gardens in the 19th century. Specific examples are Charles Darwin’s garden,^5^ and Tim Mowl and Stuart Prior’s research on the use of a garden for electrical experiments by Andrew Crosse at Fyne Court, Somerset.^6^

This paper will add to the discourse through the exploration of the gardens of two prominent and influential medical practitioners of the 18th century — John Hunter and Edward Jenner.^7^ John Hunter is perhaps best known as an anatomist, surgeon and collector — his vast collection of anatomical specimens form the basis of the Hunterian Museum at the Royal College of Surgeons in London. His first house pupil, Edward Jenner, is remembered for his work regarding the popularization and development of the smallpox vaccination. These well-known practitioners were chosen for an initial pilot study to explore how domestic gardens were used by medical practitioners specifically because of their overt use of garden space, which has been recorded by commentators and historians but not previously interrogated in depth. It will also demonstrate how the garden can be used for clinical medical practice, observations of natural phenomena and botanical experiments, and thereby provide a rich insight into domestic enquiries and concerns during the period.

## JOHN HUNTER’S GARDEN AT EARL’S COURT

In 1897, just after the destruction of Hunter’s Earl’s Court house, Stephen Paget described his emotional response:

It so expressed his [Hunter’s] work and character that the accounts of it suggest something endowed with life; and the news of its demolition, ten years ago, came like the announcement of a man’s death. It was not only alive, but highly organised, a most complex or heterogeneous structure; a farm, a menagerie, an institute of anatomy and physiology, and a villa decorated in the fashion of the period.^8^

As this was written a century after Hunter’s death, it indicates the high value Paget placed upon the location or ‘place’ of Hunter’s work. It also highlights the significance of his domestic estate in relation to his research, as well as Hunter’s status as a gentleman of fashion. As Simon Chaplin has stated, ‘Earl’s Court was personally significant’.^9^ This paper will focus specifically on the farm and menagerie aspects of Hunter’s research, and attempt to relate these areas of activity to descriptions and depictions of the designed landscape. This will build on earlier work on the history of the site, particularly by William Schupbach and the Greater London Council, and research on his museum collection by Chaplin, but will differ by focusing explicitly on the garden.

In 1764, Hunter bought two acres of land in Earl’s Court, which was then a rural village on the outskirts of London. Research commissioned by the Greater London Council in the 1980s indicates that there was already a house called Courtfield on the site and some garden features, such as the mount, which will be discussed later, may have been a material trace left over from that time.^10^ It seems that Hunter built a new house on the site in 1765 and gradually extended and added new wings to the property when he could afford it.^11^ Extracts from the Parish books included in J.J. Merriman’s description of the property demonstrate that Hunter was also extending the grounds around the house. In 1775 it was noted that he was only charged £15 for Earl’s Court; however, by 1778 the book stated that ‘John Hunter esq. House, garden £69. Ditto for part of Coleherne £30’.^12^ This suggests that he had obtained additional land, probably in 1776, which is why he was paying additional rates. He seems to have continued to buy land until his death in 1793, at which point ‘he owned the whole manor apart from Little Courtfield, a seven and half acre piece eastward of it, and the southern-half of Home Field’, much of it purchased a few months before he died.^13^ This indicates that he was creating a large agricultural estate on a piecemeal basis and that the estate itself was of a considerable size when he died.

**FIG. 1.**
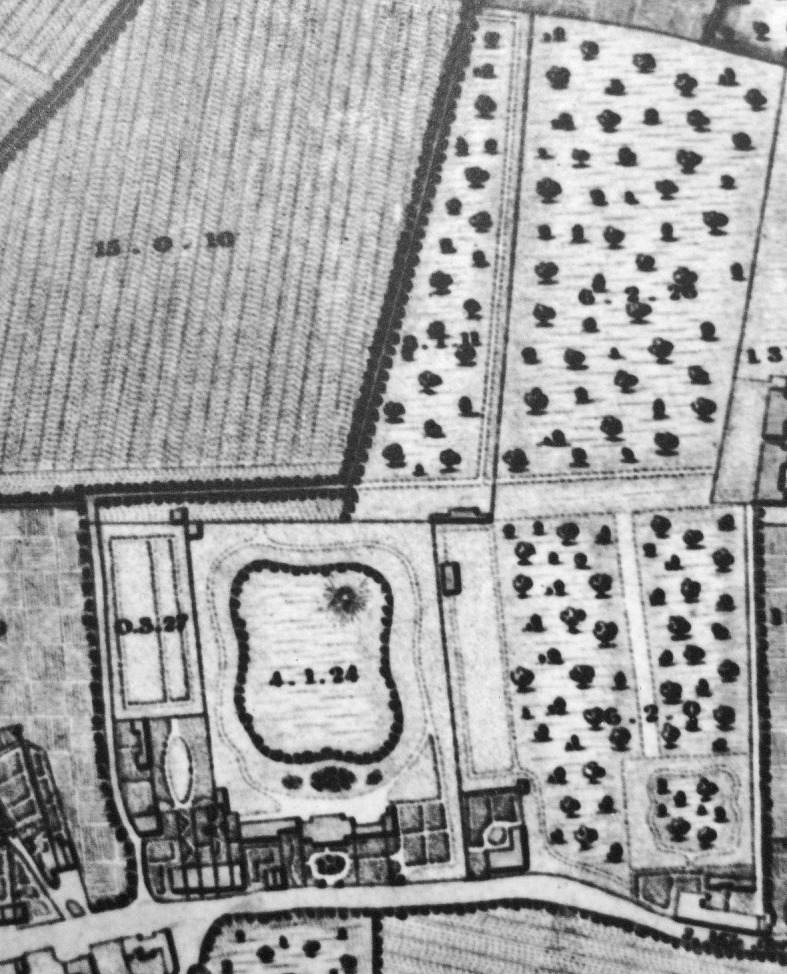
Detail of the *Plan of the Parish of St Mary Kensington*, 1822 (Kensington and Chelsea Local Studies Library)

Unfortunately, research to date has not uncovered a map of the estate created during Hunter’s lifetime. The earliest map identified is held in South Kensington local archives and was produced in 1822, 30 years after Hunter’s death (Fig. 1). However, until the 1830s the house changed hands frequently and was occupied by tenants, so it is likely that few major alterations were made to the gardens.^14^ There were some alterations of the house as ‘later plans seem to show that it was extended backwards between 1811 and 1836’.^15^ Additional written evidence from visits to the site in the 1880s, and photographs from the same decade retained in an album held by the Royal College of Surgeons archive, demonstrate that material elements within the landscape such as the ‘lions den’ or mound, remained as physical features until the destruction of the house and estate to make way for the building of the Barkston Gardens estate in 1886.

The naturalist Frank Buckland visited the site with Merriman in the 1870s. He recorded that Merriman, whose family had long been established at Kensington, assured him ‘that, according to all local tradition, the house and grounds are very little, if at all altered since the days when John Hunter lived there’.^16^ Buckland noted that there was a ‘covered cloister dug about six feet in the earth’ surrounding the house which, he surmised, would be a ‘grand place for keeping livestock’.^17^ Buckland’s desire to find any sign of Hunter remaining on the site — he was disappointed on discovering a pile of bones that they were just ‘kitchen bones’ — means that he would have seized upon anything that might be from Hunter’s time.^18^ However, his description, and the testament of Merriman, suggests that many of the physical features in the immediate vicinity of the house remained unaltered until its demolition. Although the design of the gardens immediately around the house seems to have remained relatively unaltered, the size of the estate in terms of acreage shrank quite rapidly as new housing estates were built on outlying areas of land. It is therefore difficult to reconstruct the exact scale of the Earl’s Court estate at the point of Hunter’s death.

The 1822 map suggests that there may have been connecting orchards and fields to the north and east of the house before the estate was parcelled up and sold, and these features are indicative of an agricultural or working landscape. This corresponds to the earliest description of Hunter’s gardens, which was written by Thomas Baird in 1793 following his visit to the estate. This account was published within the *General View of the Agriculture of the County of Middlesex* for the Board of Agriculture and Internal Improvement and will be discussed in more detail below.

### DEPICTIONS OF THE GARDEN

In the 1820s, the surgeon Jesse Foot created a remarkable three-volume illustrated edition of his life of Hunter, which he had originally published in text form in 1794.^19^ This combination of text and illustrations forms what Ludmilla Jordanova has described as a ‘Graingerised Version’.^20^ Jordanova also argues that when reading this work, ‘we can treat this object as a manifestation of a medical unconscious — it is full of visual associations, and actuated by a kind of fury beyond reason’.^21^ Foot, as a biographer highly biased against Hunter, uses his biographical text as a tool to discredit the late surgeon’s work. One noteworthy example is his report on Hunter’s paper on the organ of hearing in fishes, in which he stated, ‘do not these facts which I adduced, demonstrate the natural propensity, or imbecility rather, in John Hunter, stronger than any words of mine can prove it?’^22^ It is perhaps unsurprising that his description of Hunter’s house and gardens should similarly form part of his overall attempt to undermine Hunter’s reputation.

**FIG. 2.**
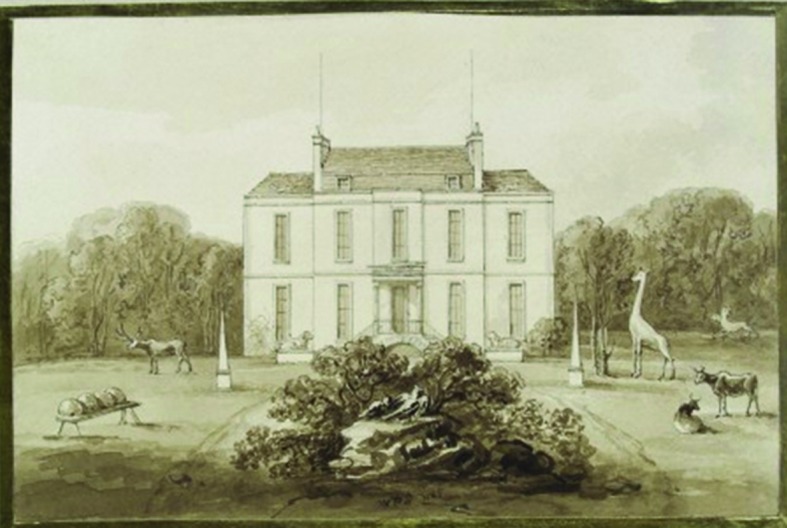
Watercolour of Hunter’s garden by Foot, 1822 (Wellcome Images).

Within the extra-illustrated version, Foot included a sketch of Hunter’s garden at Earl’s Court, which was likely to have been produced by Foot himself.^23^ This depiction is based on Foot’s textual description of the gardens and Hunter’s use of them. The illustration, which has recently been scaled up and reproduced in the Royal College of Surgeons Museum, depicts Hunter’s house as a small villa and includes bee boles, deer, a giraffe, birds chained to rocks, and what looks to be a two-headed animal in the right-hand side of the background (Fig. 2). This is a figurative illustration which attempts to depict the type of work Hunter conducted on animals and insects, much of it at Earl’s Court. Although it is an illustration which is undoubtedly based more on fantasy than fact, and was produced by someone who may never have visited Earl’s Court during Hunter’s lifetime, given the animosity between the two men,^24^ it does indicate that the domestic space of Earl’s Court was more than a place for leisure and relaxation.^25^

A close reading of Foot’s accompanying text supports the idea that his evocation of the garden was tainted with the same slanted view. Foot began his description of Earl’s Court thus:

To unbend the mind from that Tedium ... — to refresh the animal functions, half poisoned and debased, by anatomical miasma, — and to be as little as possible out of the way of the sudden calls of the surgeon, John Hunter chose a cottage at Earl’s Court.^26^

Unpicking this statement reveals Foot’s belief that the practice of anatomy was ‘debasing’ Hunter, that he was using Earl’s Court as a summer retreat rather than an active place of research, that Hunter had placed himself at a geographical distance from the calls of his practice, by implication to avoid his duty as a medical practitioner, and that the house itself was only a ‘cottage’. Here Foot is undermining Hunter as a scientist, a conscientious physician and a gentleman. This quote implies that medical practitioners were using their homes and gardens to denote their growing status as professional gentlemen, so that Foot’s downgrading of Hunter’s villa to a cottage can be seen as part of his attempt to question the surgeon’s social standing.^27^ This is similarly reflected in the illustration of Earl’s Court which is depicted without the extra architectural wings which were added over time as Hunter’s practice developed and his income increased.

In the same manner Foot stated that ‘nobody of common curiosity could have ever passed this original cottage, without being obliged to enquire, to whom it belonged’.^28^ However, research suggests that much of the livestock was at the back of the house, so would not have been seen by passers-by (although they may, of course, have heard unusual noises), and even Foot himself wrote that: ‘by observing the back of the house, a lawn was found stocked with fowls and animals, of the strangest selection in nature, — as if it had been another repository belonging to Brooks’.^29^ Foot continued:

on the sides of the area, were seen two pyramidal collections of shells, of a very contracted base, and mean height — each of them, seeming to conceal a subterranean entrance to a Golgotha. Over the front door was presented the mouth of a Crocodile, gaping tremendously wide.^30^

This is a dramatic depiction of the house and garden, which is not to say there may not be some truth to be found within it. Paget also wrote that ‘it was said that the pond is ornamented with the skulls of animals’.^31^ Skulls and shells would certainly have been available to Hunter, and the use of animal bones and shells as decorative elements in the construction of garden buildings, particularly grottos, was popular in this period. One such example of the use of animal bone is the 1770s deer-bone floor, laid in a radiating pattern, in a bath house at Wrest Park, Silsoe, Bedfordshire.^32^

### AGRICULTURAL IMPROVEMENTS AND MEDICAL PRACTITIONERS

Apart from the description by Foot, the only other contemporary depiction comes from Baird’s agricultural tract. In a section headed ‘Important Experiments’, Baird described Hunter’s estate as ‘the villa of John Hunter, the celebrated surgeon, who is trying many experiments, which may be of considerable service, both to the gardener and the husbandman’.^33^ The singling out of Hunter in a text that was interested in charting the agricultural developments of Middlesex, and in particular ‘demonstrating that the inhabitants ... are not behind any other part of the Kingdom, in zeal, for extending the knowledge and promoting the interests of their country’,^34^ confirms the theory that the role of Hunter’s garden had significance beyond his own scientific experimentation. Baird’s argument that such developments were important nationally corresponds to the research conducted by Charles Withers on agricultural improvements in 18th-century Scotland. In particular, he concludes that his research on the physician William Cullen, a contemporary of Hunter who displayed a similar interest in agricultural developments, ‘illuminates the relationships between agricultural improvement, scientific practice, and public benefit’.^35^ It is fair to say that Hunter’s work also connects these areas of interest, although, having grown up on a farm, Long Calderwood, in Scotland, he may have had a life-long, personal fascination with agricultural methods.^36^

Baird’s description of Hunter’s garden was less sensationalized, but was of course biased towards recording Hunter’s agricultural interests. He stated that Hunter was ‘very curious in plants and has in his green-houses and hot-houses a great variety of the most choice and rare productions of nature, in the collection of which he has neither spared pains or expense’.^37^ He went on to record that Hunter was experimenting with forest trees in order that:

he shall be able to direct or determine the growth of trees ... to any particular part of the trunk he may choose. For example, if from an oak, a plank is wanted of a given length and of an equal breadth at both ends ... he is of the opinion that the tree may be trained and disposed to grow in such a matter that it will yield the plank of the exact dimensions required.^38^

This narrative concerning experimentation with tree growth is confirmed by Chaplin, who notes that:

a notice of the demolition of the house in 1886 mentioned some of the physical evidence of his researches, noting that many of the mature trees bore ‘marks of his insatiable desire for mixture’, including ‘a rough-skinned oak, with smooth-skinned branches grafted on to it’.^39^

This interest in trees corresponded to a general interest in agricultural improvements and the use of landscape features such as forests for economic purposes as well as aesthetic appreciation in the 18th century. For example, Alexander Hunter (no obvious relation to John) was another 18th-century medical practitioner whose interest in trees led to him to publish, in 1776, a revised version of John Evelyn’s *Silva.* This was seen as a significant work and cited as one of the reasons for his successful nomination to membership of the Royal Society.^40^

As the agricultural historian G.E. Fussell has stated, ‘all doctors were botanists in the eighteenth century’,^41^ and studying botany was an important element of medical education during this period. The importance of the connections between botany and medicine is illustrated by the fact that the first president of the Linnean Society of London in 1788 was a medical practitioner, James Edward Smith. There were also interconnections between botanical, medical and agricultural knowledge. The apothecary William Curtis founded a subscription botanic garden in Brompton in 1778.^42^ In his *Proposals for Opening by Subscription a Botanic Garden to be Called the London Botanic Garden*, he states that it would be designed for the ‘use of the Physician, the Apothecary, the student in Physic, the scientific Farmer, the Botanist (particularly the English Botanist), the lover of Flowers and the Public in General’.^43^ He particularly saw the value of botanical knowledge to both medical practice and agriculture. In relation to medical practice, he noted the decline in the use of local plants in remedies, but felt that practitioners should be able to recognize those that were still in use. He wrote:

although new discovered chemical remedies, and foreign drugs, may have justly superseded many of our English plants, yet a great number are still retained in our Phamacopoea [*sic*], and many possess very poisonous qualities: to be acquainted with these at least, is the duty of every one, that takes on himself the important character of guardian of the healths of mankind.^44^

He also claimed that botanical knowledge ‘may be applied with as much advantage to agriculture as to any other science’, and went on to say that he hoped the garden will ‘become productive of national utility’.^45^

This interest in plants in relation to concerns regarding agricultural improvements seems to have been common amongst medical practitioners during this period. For example, in 1809 Joseph Banks, President of the Royal Society, wrote Smith the following letter:

I Send you a few Roots & Some Runners also of Grass Call’d in Irish Fiorin which Dr Richardson has publishd much about with the intention of persuading us here that it [is] a most invaluable addition to our husbandry & will at Least if adopted double the population of the Island by increasing the food for winter ... pray Plant in your Garden or in that of Some friend that we may when it Comes into fructification Conferr about its right name which I Suspect to be agrotis Stolonifera thriving better under the damp atmosphere of Ireland than it will do in the dry one of Spring Grove & Norwich.^46^

This demonstrates that there was a network formed of doctors through the transmission of plant material and that medical practitioners were involved in attempts to increase the food supply of the population, alongside gentleman scientists and landowners. Medical practitioners formed a sub-section of larger networks between gentleman scientists and others during this period. For example, Banks wrote a few months later to the agricultural reformer, Arthur Young, about the same species of grass, which establishes that the network also included prominent agriculturalists. The letter above highlights the wide use of domestic gardens for such trials and experimentation and establishes that Hunter was not a singular example of this use of the domestic garden.

Fussell also quotes from an anonymous text published in 1760, which argued that, in order to educate farmers:

those who will make improvements should be set in ‘convenient farms almost in every district in the country’, ‘and called upon the physician to use the leisure spared from raising wholesome food for the preservation of health, and from cultivating herbs necessary to cure disease, in improving manures and adapting plants to proper soil’.^47^

This suggests there was an expectation on the part of the writer that medical practitioners should use their leisure time to study soil, because better food supplies and botanical remedies were reducing the workload of physicians. There is perhaps an implied belief that medical practitioners had specialist knowledge in both botany and chemistry, so were ideally placed to conduct this work. This is an area which deserves future exploration.

In line with other medical practitioners of the period, and no doubt using his time in a way the writer above would have approved, Hunter was also conducting trials regarding different types of compost. Baird records how Hunter had developed an alternative to using peat, which was expensive and for which he had to rely on others to obtain, for his green- and hot-house plants. Using his observational skills, Hunter determined that as ‘this turf [peat] was no other than the roots of vegetables rotted, something else might be substituted, which would answer equally well for raising his plants’.^48^ Baird went on to describe how Hunter decided oak bark would work just as well:

for a trial he caused a quantity of it, after having served the purposes of the hot-house, to be buried in this exhausted state, in the earth for upwards of eight years, when it was taken up and being used in the place of the turf, he found it answer in every respect and continues to use nothing else.^49^

The doctor, Francis Home, won a prize for his treatise on soil,^50^ and Alexander Hunter’s *Georgics*, which was a central reason for his election to the Royal Society,^51^ similarly concentrated on soil. So prevalent was this interest in soil and compost amongst medical practitioners that Jenner was also conducting experiments in his garden at Berkeley. On 5 June 1787, Jenner writes to Banks describing various experiments he has been doing since 1780 on whether blood was a useful additive that would increase soil fertility.^52^ According to this letter, in February,

a small quantity of the Serum of human blood was pour’d over about a square foot of grass on a grass-plot. Three sprinklings were given at the distance of a fortnight each, and the whole quantity applied was the serum contain’d in forty Ounces of blood.^53^

By April, he records ‘that the effects it has produc’d on the vegetation of the grass is astonishing. It is beautifully green & thick & has sprung up several inches while the surrounding grass has but just begun to shoot’.^54^ The use of blood had a less positive outcome on polyanthus plants as, ‘at about the time when the flower-stems (which were uncommonly vigorous) were push’d up to about half their height, they suddenly wither’d away & died’.^55^ Similarly, the peach trees which did best were those which were fertilized with animal manure. He conducted variations of the same experiment on currant trees and mustard seed, and it is fair to assume that all these trials were conducted in his garden at Berkeley.

As well as his work on plants and compost, Hunter also conducted experiments on animals on his estate. Using Hunter’s papers and other descriptions, Paget compiled a list of animals which he believed were kept at Earl’s Court:

in a field facing his sitting room was a pond, where he kept for experiment his fishes, frogs, leeches, eels and river-mussels ... The trees dotted about the grounds served him for his studies of the heat of living plants, their movements and their power of repair. He kept fowls, ducks, geese, pigeons, rabbits, pigs, and made experiments on them; also opposums, hedgehogs and rare animals — a jackal, a zebra, an ostrich, buffaloes, even leopards; also dormice, bats, snakes and birds of prey.^56^

Although this was compiled a century after Hunter’s death and some animals may have been kept in locations other than Earl’s Court, it is worth noting that many of the creatures listed are domestic animals. It would also seem that his interest in the production of heat by animals and vegetables seems to have been a strong factor in the animals and plants obtained, and the subsequent experiments conducted.^57^ Hunter wrote that he conducted some of these experiments to ‘ascertain whether vegetables could be frozen, and afterwards retain all their properties when thawed, or had the same power of generating heat with animals’.^58^

Baird certainly had more of a pastoral slant on the scenes that he witnessed in 1793, although as Hunter was 65 and very successful this may represent a point in Hunter’s life when he had an increased amount of time and money to spend on developing the estate. Baird recorded that:

the variety of birds and beasts to be met with at Earl’s Court ... is a matter of great entertainment. In the same ground you are suprized to find so many living animals, in one herd, from the most opposite parts of the habitable globe. Buffaloes, rams and sheep from Turkey, and a shawl goat from the East Indies, are among the most remarkable of those that meet the eye.^59^

Although Baird stated that they are a matter of entertainment, thereby implying the animals were an element of spectacle within the landscape, the most exotic that he described on this visit were still animals that were bred first and foremost for wool and meat production. These domestic beasts are the most prevalent in Baird’s description and the vaults built around the house (also described by Buckland) were depicted as follows: ‘Mr Hunter built his stables half underground; and that he also had vaults in which he keeps his cows, buffaloes and hogs’.^60^ It is evident from this portrayal that much of Hunter’s interest in these animals relates to cross-breeding for agricultural purposes. Although Baird focused on the agricultural aspects of Hunter’s work, this does suggest an agricultural estate rather more than a menagerie, at least in the 1790s.

Hunter’s agricultural interest was echoed by other medical practitioners of the time. Cullen was certainly conducting agricultural experiments on his own farm and that belonging to his brother near Glasgow. The findings from these experiments were translated into lectures, which he presented to students, many of whom were presumably studying medicine. This demonstrates both his interest in the scientific basis of agricultural production and the importance of its dissemination. As Withers has noted, his ‘involvement in agricultural improvement and particularly his lectures on agriculture, agricultural chemistry and the bases to plant nutrition suggests Cullen to have been an important individual figure in agricultural circles in eighteenth-century Scotland’.^61^ This provides further evidence of the connections between medical practitioners, the subjects of chemistry and botany — both subjects were taught at medical school — and the production of scientific knowledge on their domestic estates regarding agricultural improvements. There are evidently crossovers here between medical knowledge, natural history and attempts at agricultural improvements.

Although this paper focuses on the activities of medical practitioners, discussions concerning potential agricultural improvements were widespread. The increasing pace of enclosure in the 18th century meant that landowners appropriated land for forestry, arable crops and animal production, and therefore often took an interest in the economic production of their land. As Tom Williamson has stated, however, there was a blurring between economic necessity and demonstrations of status:

everything in the landscape of the park had complex, overlapping functions. The animals grazing on the turf were more than lawn-mowers, more than machines for producing meat and wool. Members of the aristocracy and gentry alike took as keen an interest in the selective breeding of livestock as they did in the improvement of their horses and foxhounds.^62^

This places Hunter within a social group whose status as landowners meant a shared interest in using findings from scientific experimentation to increase their own personal profits. As Michael Brown has demonstrated, ‘agricultural knowledge was polite knowledge’ and could be used to build influential kinship networks and raise the societal status of physicians.^63^

### BOTANY AND NATURAL HISTORY

As well as the agricultural investigations recorded by Baird, Hunter also conducted other botanical and natural history experiments, as recorded within his letters and published articles. Notable examples that were conducted at Earl’s Court include an attempt to culture pearls in the garden pond,^64^ and his research on bees. His observations on bees perhaps best demonstrate his close observation of the natural world within his domestic space — it can be argued that this skill is what set him apart from other surgeons of the time.

**FIG. 3.**
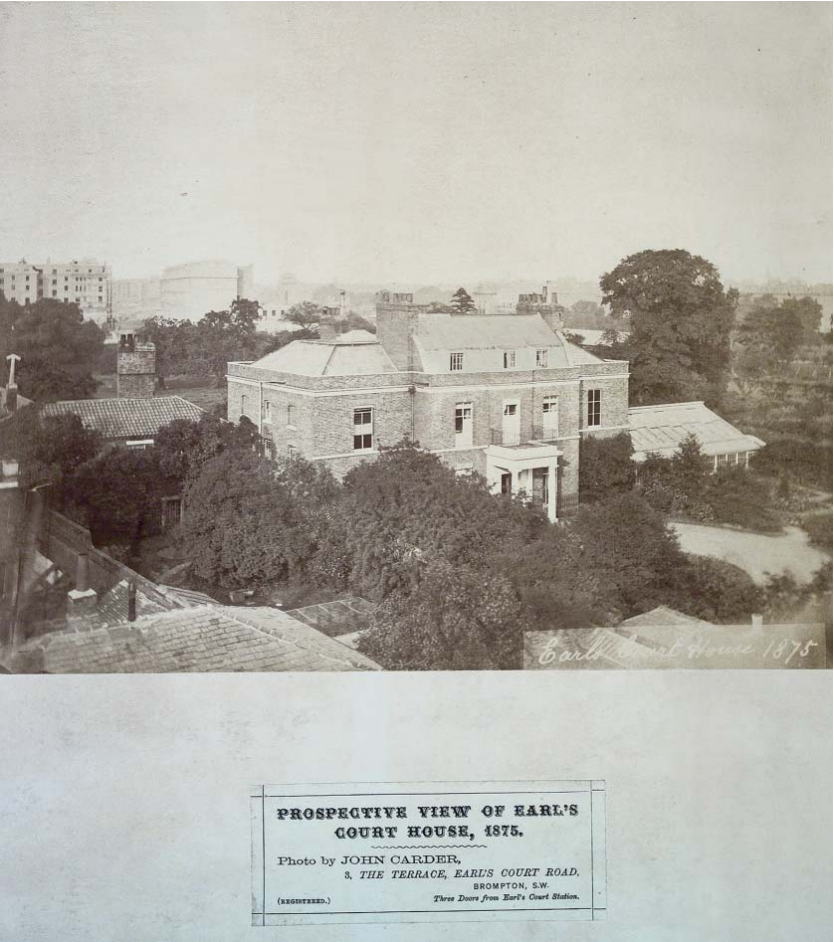
Photograph of Earl’s Court house taken just before its demolition in 1875, showing the conservatory in which Hunter kept his bees to the right of the house (Hunterian family album, Royal College of Surgeons Archive).

Hunter’s specially constructed hives were placed within the conservatory adjoining the house at Earl’s Court (Fig. 3), which would have enabled him to conduct his detailed observational work over a period of time. Similarly, having plants at close quarters enabled his research. Hunter’s method of working when studying the *Mimosa pudica* demonstrates this:

in order to have the greatest part of the day before me, I began my experiments at 8 in the morning, while the leaves were in full expansion, and I continued them till 4 in the afternoon, as longer would not have been just, for they begin to collapse of themselves between 5 and 6 o’clock.^65^

The use of the domestic space would allow for this continual monitoring and observational work in any leisure time around his medical practice conducted in the centre of London.

Hunter’s paper on bees was the last work he contributed to *Philosophical Transactions* in 1792. In order to conduct research into this subject he had glass hives constructed to his own specification so that they had:

different panes of glass, each pane opening with hinges so that if I saw anything going on that I wished to examine more minutely or immediately, I opened the pane at this part and executed what I wished, as much as was in my power.^66^

He recorded his observations as follows:

when I saw some operations going on the dates or periods of which I wished to ascertain, such as the time of laying eggs, of hatching, &c. I made a little dot with white paint opposite to the cell where the egg was laid and put down the date.^67^

His close observational work is clearly demonstrated by his detailed attention to the sound produced by bees:

Bees may be said to have a voice ... But they produce a noise independent of their wings; for if a bee is smeared all over with honey, so as to make the wings stick together it will be found to make a noise, which is shrill and peevish. To ascertain this further, I held a bee by the legs, with a pair of pincers; and observed it then made the peevish noise, although the wings were perfectly still: I then cut the wings off, and found it made the same noise. I examined it in water, but it then did not produce the noise, till it was very much teased and then it made the same kind of noise; and I could observe the water, or rather the surface of contact of the water with the air at the mouth of an air-hole at the root of the wing vibrating ... I have observed that they, or some of them, make a noise the evenings before they swarm, which is a kind of ring, or sound of a small trumpet: by comparing it with the notes of the piano forte, it seemed to be the same with the lower A of the treble.^68^

This interest in bees can perhaps be related to his agricultural interest in animals, compost, silkworms and trees — they were all important in relation to the economic value of an estate.

### THE LION MOUND

One material piece of evidence which relates to a more exotic tale of animal husbandry is the mound in the garden to the back of the house. The date of the construction of the mound is unknown. As Earl’s Court house was built on the estate which had belonged to Courtfield some of the physical landscape features may relate to the previous residence, which appears to have been demolished by the time Hunter purchased the land.^69^ Accounts suggest that the gardens of Courtfield were extensive and the creation of mounds was certainly more popular in the 17th than the 18th century. According to the *Survey of London*, in 1705 the occupant of Courtfield, John Bowack:

... described it as ‘but lately Built after the Modern Manner, and standing upon a Plain where nothing can intercept the Sight looks very Stately at a Distance, [the] Gardens are very good ...’. ... Descriptions in the 1690s emphasize the garden, mentioning ‘walkes’, ‘waterworkes’, ‘engines for water’, a summer-house and great garden gates with a ‘sweep’ of ground on the outer or eastern side of them.^70^

This description portrays an ornamental landscape in which a viewing mound would not be out of place. What is more certain is that the mound was constructed, at least in part, as an aesthetic garden feature as it had an ornamental ‘battery’ on its summit. According to Paget, ‘on top of the mound was a little rampart of bricks and tiles, making a toy fortress of it’.^71^

**FIG. 4.**
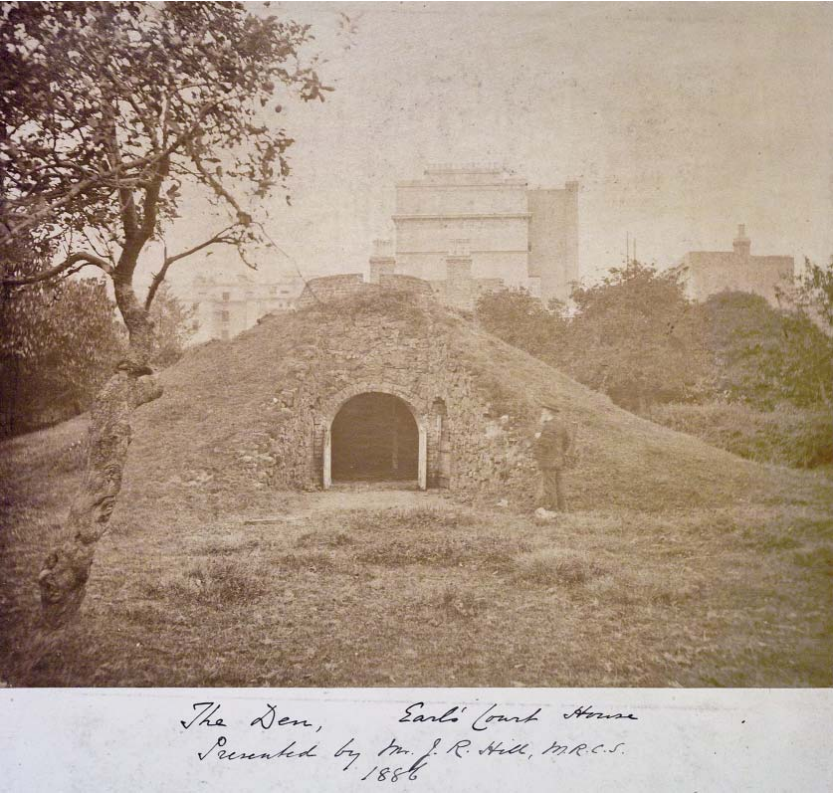
Photograph of the mound at Earl’s Court in 1886 (Hunterian family album, Royal College of Surgeons Archive).

**FIG. 5.**
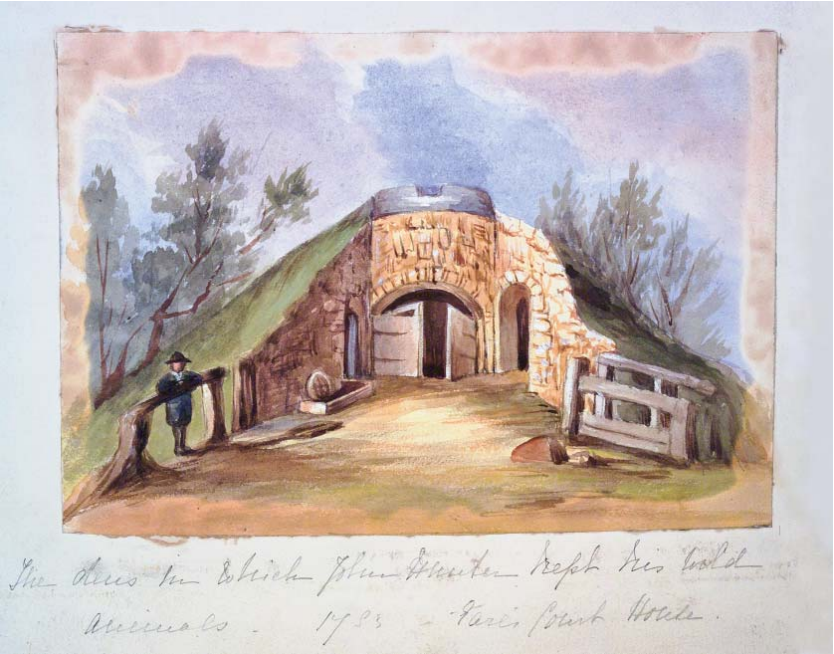
Watercolour of the mound at Earl’s Court with a pencil date of 1783, showing the mound as a pastoral feature (Hunterian family album, Royal College of Surgeons Archive).

Whether or not the mound was an artefact of a previous age or built by Hunter himself, we know that he utilized it and that it remained in the garden until the demolition of the house in the 1880s (Fig. 4). There is a watercolour in the Hunter family album (Fig. 5) which suggests that it had a pastoral feel and that during Hunter’s time the mound was used as an animal pen, even if it was not originally designed with that use in mind. This type of mixed use fits the style of 18th-century landscape design known as the *ferme ornée* or ‘ornamented farm’, where utilitarian buildings, such as cowsheds, could also be attractive features acting as eye-catchers or decorative structures within the landscape.^72^ So Hunter’s use of a physical feature for aesthetic and pastoral purposes was in keeping with the aesthetic taste of the period. The Greater London Council Survey published in the 1980s refers to it as a ‘mound containing vaulted byres for the larger animals’.^73^ The mound was referred to as the ‘lion’s den’ by Buckland in 1875 and, although there is no record of Hunter housing a lion, there is a narrative regarding Hunter and the keeping of leopards at Earl’s Court. As Wendy Moore relates: ‘the leopards once broke free from their chains and ran into the yard where they attacked the dogs. ... Somehow he managed to catch the animals and get them back in the den’.^74^ However, whether the den is in fact the mound or some other feature is unclear and, according to Schupbach, Stephen Pasmore has suggested that the mound was actually used to keep Hunter’s buffaloes.^75^

The existence of the mound as late as the 1880s establishes that the landscape at Earl’s Court was modified, but not entirely changed during the 19th century. Therefore the maps and photographs produced after Hunter’s death offer us a glimpse of the material surroundings of the villa of Earl’s Court — a location which appears to have been a place of agricultural and botanical experimentation.

## EDWARD JENNER AT THE CHANTRY, BERKELEY, GLOUCESTERSHIRE

In comparison to Hunter’s activity and experimentation, Jenner’s house at Berkeley (now a museum known as Dr Jenner’s House) was surrounded by a more leisurely landscape intended for gentlemanly contemplation, although even here the garden was a site of scientific and medical activity. Jenner was a Romantic gentleman and the founder and first president of the Cheltenham Literary and Philosophical Society. Unlike Hunter, his status from birth was of a man with a modest private income and connections with the minor landed gentry, but the two men shared a lifelong interest in natural history. Jenner was Hunter’s first house pupil and the two men maintained a correspondence and friendship throughout their lives.

Although internationally renowned for his work on smallpox, Jenner had wide-ranging interests in the natural world.^76^ In 1798 he was elected to fellowship of the Linnean Society, he was a member of the Royal Geological Society and was conferred membership of the Royal Society for his observational work on cuckoos. According to his first biographer, John Baron:

his knowledge of the economy of plants and animals, and his vigilant attention to all the varied forms and properties of surrounding objects, supplied him with an inexhaustible fund of analogies and imagery, which alike animated and adorned every subject that he touched upon.^77^

### THE TEMPLE OF VACCINIA

**FIG. 6.**
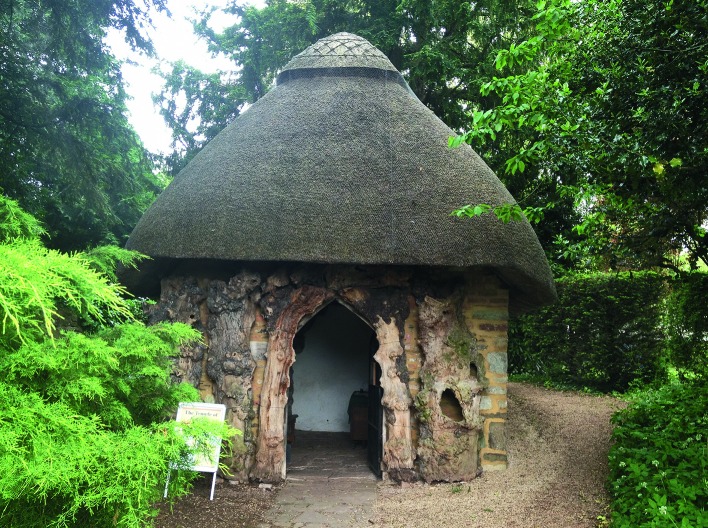
Temple of Vaccinia (photograph, C. Hickman, 2013).

Undoubtedly the most interesting physical structure in Jenner’s garden is the summerhouse known as the Temple of Vaccinia, in which Jenner conducted free vaccinations (Fig. 6). Unlike Hunter’s garden, which seems to have been primarily used as a place for research, Jenner’s garden appears to have been an aesthetically stylish landscape which was then used for his fertilization experiments and medical practice. A letter that has recently come to light confirms that the Temple was originally constructed as a place of leisure in the manner of most garden buildings of the period. On 19 May 1804, Mr Joyce wrote to Dr Lettsom and described his visit. He arrived as Jenner was sitting down to breakfast:

This parlour in which we were sitting look’d into an agreeable lawn, on one side of which ran a walk ... I had observed during our conversation a great number of females with children in their arms or by their side, passing down this walk ...; and I could not forebear interrupting the conversation to enquire of my friend, what it meant. It has been the custom for some time, said he, to set apart one morning in the week for inoculating the poor ... In the midst of those trees is a small mansion built in the cottage stile [*sic*]. It consists of one room only and was erected for the purpose of giving a rural appearance to that part of my garden. I have lately converted it into a place of utility — and the people who come to be inoculated assemble there and wait until I come among them. It is for this reason, I have given my little cottage the name of the Temple of Vaccinia.^78^

This makes it clear that the building was originally ornamental and later adapted in its use, and that the term Temple of Vaccinia was conferred upon it by Jenner before 1804.

The building itself, and possibly the garden, seems to have been the vision of the Revd Ferryman. In 1821 Thomas Fosbroke recorded how Jenner was ‘acquainted with a Clergyman of very original and surpassing taste in two particular departments of picturesque gardening, of exceeding difficulty, especially the first, *viz* exquisite informal primrose tumps and perfect rustic work’.^79^ He continued:

The open side of it is covered by an irregular primrosed tump, surmounted by a branchy decayed stump, like a classical trophy, supporting a flaunting honeysuckle. ... One might suppose it the residence of a Faun or a Dryad, or an Arcadian Deity. The furniture is a rustic chair, composed of the malleable and elastic stems of ivy, tastefully reticulated. The approach to the cottage is, in the scenepainting style, through insulated trees, not close enough for wood, only joining at their heads.^80^

This description conjures the impression of a gentleman’s garden which has been designed according to the taste of the time. The debates regarding the ‘Picturesque’ style in relation to gardening were being played out at this time around the Wye Valley, which is geographically not that distant from Berkeley. Baron himself makes the link between Ferryman’s gardening design and the work of Uvedale Price, who was one of the central figures attempting to define what ‘Picturesque’ meant: ‘I do not know if he ever read the elegant work of the late accomplished Sir Uvedale Price, ... but there is a relationship between their conceptions, and a truth in their practical elucidations which stamps as brothers in the same family of genius’.^81^ This firmly places the buildings within the style lexicon of the period.

The Revd Ferryman himself was an intriguing figure. There is not space to discuss him in detail here, but Hugh Torrens’ entry in the *Oxford Dictionary of National Biography* gives an account of his varied life, which included occupations such as curate, brewer, natural historian, and, according to Jenner, also a garden designer and architect.^82^ In December 1817, when Ferryman returned from his second trip to Canada, Jenner wrote to Thomas Pruen:

what a strange jumble of intellect does that unfortunate man possess. How much he has mistaken himself, & put that in front which should have been in the background. He is pre-eminent (in my opinion) as a Landscape Gardener & by pursuing this for the benefit of others, he might have enrich’d himself but he must become an architect & be hanged to him, ruin himself & those who were heedless to employ him.^83^

This follows an earlier letter in 1813 to his son, in which he wrote,

I have not seen or heard anything of Mr Ferryman in this part of the World, and of course am still without the Lettuce Seed. Having obtain’d a promise of Garden Seeds from the South of Spain & from Genoa, I am in hopes they may arrive time enough for sowing.^84^

These letters indicate that, although he has tried Jenner’s patience, Jenner still holds him in high regard as a landscape designer and as someone who can obtain interesting seeds for him. Like Hunter, Jenner was also interested in novel plants and one letter between himself and Thomas Paytherus in 1810 mentions his offer of a gift of white strawberries and Jenner’s desire to obtain *transparent apples* [Jenner’s emphasis].^85^

The texts also establish that the original Temple was simply a summer house in the Picturesque style — one which appears to fit the 18th-century aesthetic of a rustic retreat which could be used for contemplation. This type of construction is illustrated in Thomas Wright’s *Universal Architecture*, published in two volumes in the 1750s: *Grottos and Arbours*. These volumes contained Wright’s designs for garden retreats. The rustic style is most evident in the* Arbours* volume with designs ‘for a Hut or Hovel-kind, chiefly designed for a shelter’d solitude’ and ‘a Druid’s Cell, or Arbour of the Hermitage Kind, purposely designed for a Study or Philosophical retirement’.^86^ This is not to suggest that Wright had anything to do with Jenner’s building, but the rusticated style would be associated in particular with contemplation. As Gervase Jackson-Stops has established, hermitages were ‘always primitive and rustic, made of boulders, roots or bark, and with thatched or turfed roofs, [and] just as popular with gentry of more modest means’.^87^ A similar hut designated as a ‘hermitage’ was built by Gilbert White of Selborne in the 1750s, which provides further evidence for the relationship between the style and its use. As it was depicted in paintings, including a 1777 watercolour by Samuel Hieronymous Grimm with Henry White, Gilbert’s brother, dressed as the hermit, this rustic building might have provided some inspiration, particularly given the mutual interest of Jenner and White in natural history.^88^

**FIG. 7.**
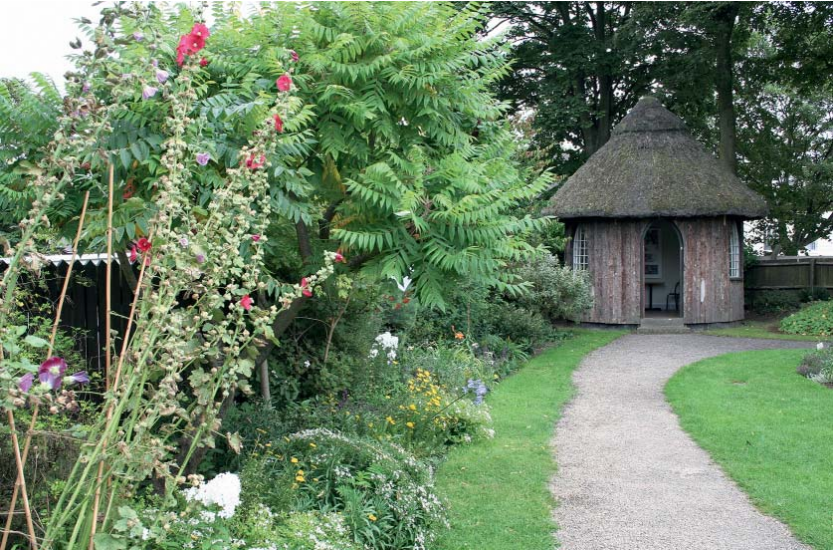
The restored hermitage at Soho (with thanks to Timothy Mowl)

Jenner’s Temple also bears a striking resemblance to a hermitage constructed by Matthew Boulton on his Soho Estate (Fig. 7). This was similarly a thatched rustic hut, and intriguingly there is a connection between Ferryman and Boulton, which leads one to wonder if Ferryman used the example at Soho as a model. One of the many activities for which Ferryman developed a reputation, was the design of a lock used at both Gloucester Gaol and Gloucester Infirmary in the 1790s. Torrens states that Matthew Boulton was involved in the manufacture of these popular locks, and ‘as late as 1807 Boulton and Watt were quoting for steam engines through Ferryman’.^89^ A conjectural plan of the Soho estate in 1794, based on extensive archival research by Phillada Ballard, clearly shows that a walk between the House at Soho and the Manufactory would have taken any visitor past the rustic hermitage.^90^ Assuming that Ferryman visited Soho, which, given the business relationship between the two men, seems likely, he would have seen the hermitage. From this, admittedly conjectural argument, there arises the possibility that Ferryman copied, or at least took inspiration from the hermitage at Soho. The examples of rustic buildings discussed here were all owned by gentlemen with scientific and natural history interests and this suggests that Jenner was a Romantic gentleman who used his garden foremost to display his aesthetic taste. This space was then adapted and utilized as a scientific and medical space — scientific in relation to his plant experiments, and clinically appropriated for the execution of vaccinations.

The secondary use of the Temple as the space in which Jenner vaccinated the poor against smallpox has led to the building’s greater historical significance. The reception of the garden building as a place of medical importance has developed out of Jenner’s seminal work on smallpox and his dissemination of the vaccination methodology as a form of preventive medicine. The garden, therefore, is the place of his significant medical activity and the Temple forms a historical focus. In 2011 the Temple was re-launched after extensive restoration and it is evident that both the place and the material structure of the summerhouse still hold meaning and significance in the 21st century. The multi-layered deployment of the garden space for both leisure and endeavour at Berkeley was perhaps more common amongst other medical practitioners of the period than Hunter’s intensive ‘biological research station’,^91^ although more extensive research needs to be conducted for this to be confirmed.

## CONCLUSION

The gardens of Hunter and Jenner reveal the importance of ‘place’ in medical and scientific practice in the 18th century. The designed landscapes have multi-layered meanings and provide a lens through which to explore ideas of status, knowledge production and the life of medical practitioners within the domestic sphere. In particular, Hunter’s garden sits somewhere in the permeable border zone between the laboratory and the field as theorized by Kohler.

This paper has also demonstrated that there are strong connections between botany, medical practice and agriculture in this period. This may in part be facilitated by the medical education of the time. As John Pickstone, has stated:

botanical gardens developed out of herbal gardens, set up by Universities or by medical guilds. Chemistry, too, was in part a matter of improving other trades and crafts, including agriculture. By 1700 in Holland, and 1750 in Edinburgh, much of this new chemistry, botany and anatomy was included in the education of physicians.^92^

There was also a cross-over in the methods and interests used in medical practice and botanical work. As Pickstone has also elucidated, the interest in collecting medical case studies was mirrored in botanical classification:

This kind of medicine drew on classical traditions, especially the writings attributed to Hipppocrates. Its chief British representative was Thomas Sydenham, a reformer of medicine who moved in the same circles as the physician and philosopher John Locke, and who shared that interest in plants and classification which became so seminal for so many eighteenth-century ‘savants’.^93^

Hunter was particularly interested in classification, and his comparative anatomy specimen collection represented his own attempt to find connections between different elements of the natural world. His work is perhaps the exemplar of Pickstone’s assertion that the anatomical work of surgeons in the late 18th century ‘still operated within the hegemony of a natural-historical, biographical medicine’, and I would suggest that this approach permeated into his use of the gardens at Earl’s Court as a place where he could apply this to the natural world more generally.

There is also perhaps evidence of a move towards the experimental approach which became popular from the 19th century. The garden cannot be a place-less laboratory as there are natural variations to contend with, ranging from soil to weather conditions. However, some elements can be controlled and Hunter’s glass hives in the conservatory can be seen as an attempt to create what we would now class as laboratory conditions. They are used predominantly for observation, but it is a move away from natural historical research in the field. Similarly, Jenner’s trials with different fertilizers can also be seen as a precursor to the experimental approach.

Finally, the use of the garden as a multi- layered space by Jenner also suggests that our perception of the utilization of designed landscapes in the 18th century needs to take on similar multi-layered approaches. As Livingstone has stated, ‘between the archive and the field, the world of the museum and the world of nature, stands the garden’.^94^ In the case of Hunter, the garden really was a place between nature and the museum as the living specimens at Earl’s Court were often transferred ‘in the form of preparations to be displayed in the museum of Leicester Square’.^95^ In the examples of Jenner and Hunter the garden can be ornamental in form, a working farm, a place for clinical medical practice (vaccination) and a space for observation and experimentation — all at the same time.

## NOTES

^1^ In particular, see Mowl 2000 and Dixon-Hunt 1976.

^2^ Exemplary examples of this are Kohler 2002 and Naylor 2010.

^3^ Livingstone 2003.

^4^ Elliott 2010.

^5^ Boulter 2008.

^6^ Mowl & Prior 2009.

^7^ The lives and medical practice of both men has already been covered extensively elsewhere, so will not be discussed in detail here.

^8^ Paget 1897, 85–6.

^9^ Chaplin 2009, 230.

^10^ Greater London Council 1986, 196–8.

^11^ Merriman, 1886, 3.

^12^ Merriman, 1886, 3.

^13^ Greater London Council 1986, 196.

^14^ After Hunter died the house was purchased by the Duke of Richmond for his ‘housekeeper’ and mistress, Mrs Bennett. She then leased it to the Earl of Albemarle, who lived there until 1810. There was then a gap in recorded ownership until it became a private lunatic asylum in the 1830s.

^15^ Greater London Council 1986, 197.

^16^ Buckland 1875, 315.

^17^ Buckland 1875, 315.

^18^ However, there were human bones secreted on the site, as builders discovered burial pits containing human remains in the 1890s: Chaplin 2009, 63, fn. 93.

^19^ Foot 1822.

^20^ Jordanova 1997, 112.

^21^ Jordanova 1997, 113.

^22^ Foot 1794, 272–3.

^23^ Jordanova states that she believes the accompanying paintings in the text were made by Foot: Jordanova 1997, 112.

^24^ See Bevan 2004.

^25^ Schupbach 1986, 352.

^26^ Foot 1794, 240–1.

^27^ For more on the use of land ownership in relation to social standing and professionalization in this period, see Brown 2011.

^28^ Foot 1794, 241.

^29^ Foot 1794, 241. His reference to Brooks here is made in relation to Joshua Brooks, who was also an anatomist with an associated menagerie. However, in contrast to Hunter’s fairly rural retreat, research by Tim Knox has discovered that Brooks’ place was in the centre of London and definitely designed for spectacle and entertainment: Knox 1997–98.

^30^ Foot 1794, 241.

^31^ Paget 1897, 87.

^32^ English Heritage n.d., List Entry Number 1113790, <http://list.english-heritage.org.uk> [accessed 3 August 2012].

^33^ Baird 1793, 38.

^34^ Baird 1793, 8.

^35^ Withers 1998, 156.

^36^ Moore 2005, 45–6.

^37^ Baird 1793, 38.

^38^ Baird 1793, 38.

^39^ Chaplin 2009, 230.

^40^ Brown 2011, 51–8.

^41^ Fussell 1950, 130.

^42^ For more on Curtis and the London Botanic Garden, see Clark 2009–10.

^43^ Curtis 1778, title page.

^44^ Curtis 1778, 6.

^45^ Curtis 1778, 7.

^46^ Chambers 2007, **5**, 505.

^47^ The Anonymous text was called A Dissertation on the Chief Obstacles to the Improvement of Land and Introducing Better Methods of Agriculture throughout Scotland, quoted in Fussell 1950, 35.

^48^ Baird 1793, 39.

^49^ Baird 1793, 39.

^50^ Home 1756.

^51^ See Brown 2011, 51.

^52^ With thanks to Andrew Legg for drawing my attention to this letter.

^53^ Miller 1983, 4–5.

^54^ Miller 1983, 4–5.

^55^ Miller 1983, 4–5.

^56^ Paget 1897, 87.

^57^ For a detailed account of the biology experiments Hunter conducted, see Hulke 1895, 405–10.

^58^ Hunter 1786, 177.

^59^ Baird 1793, 39.

^60^ Baird 1793, 40.

^61^ Withers 1998, 148.

^62^ Williamson 1995, 121.

^63^ Brown 2011, 56.

^64^ Hunter writes about this in a letter to Joseph Banks, reprinted in Buckland 1875, 327.

^65^ Hulke 1895, 405.

^66^ Hunter 1792, 132.

^67^ Hunter 1792, 132.

^68^ Hunter 1792, 182–3.

^69^ Greater London Council 1986, 197.

^70^ Greater London Council 1986, 197.

^71^ Paget 1897, 87.

^72^ Williamson 1995, 122–4.

^73^ Greater London Council 1986, 197.

^74^ Moore 2005, 293.

^75^ Schupbach 1986, 351.

^76^ For more on Jenner’s work relating to smallpox, see Williams 2010.

^77^ Baron 1838, **1**, 18.

^78^ With thanks to Dr Jenner’s House staff for bringing this to my attention: the letter is held in the New York Academy of Medicine, MS 1277.

^79^ Fosbroke 1821, 226–7.

^80^ Fosbroke 1821, 226–7.

^81^ Baron 1838, **2**, 298

^82^ Torrens 2008.

^83^ Miller 1983, 80.

^84^ Miller 1983, 89.

^85^ Miller 1983, 65–6.

^86^ Wright, 1755, no page numbers.

^87^ Jackson-Stops 1992, 77–8.

^88^ Jackson-Stops 1992, 77–8.

^89^ Torrens 2008.

^90^ Ballard *et al*. 2009, 4.

^91^ Schupbach 1986, 351.

^92^ Pickstone 2000, 68.

^93^ Pickstone 2000, 68.

^94^ Livingstone 2003, 48.

^95^ Chaplin 2009, 230.

